# Therapist self-disclosure: a systematic review and brief person-centered framework for clinical practice and training

**DOI:** 10.3389/fpsyg.2026.1869955

**Published:** 2026-07-31

**Authors:** Danielle Jeffery

**Affiliations:** Faculty of Behavioural Sciences Yorkville University, Fredericton, NB, Canada

**Keywords:** congruence, cultural broaching, cultural competence, person-centered therapy, psychotherapy training, Rogerian core conditions, therapeutic alliance, therapist self-disclosure

## Abstract

**Introduction:**

Therapist self-disclosure (TSD) is a widely used yet clinically complex intervention associated with both therapeutic benefit and potential harm. Existing literature suggests that TSD can strengthen the therapeutic relationship, enhance client engagement, and support culturally responsive practice. However, uncertainty remains regarding when, how, and why therapists should disclose personal information in clinical settings.

**Methods:**

This systematic review synthesized contemporary empirical literature examining the effectiveness, risks, benefits, and cultural considerations associated with TSD. A PRISMA-guided review process was employed, and findings were analyzed using both inductive and deductive thematic analysis. The review also examined the continued relevance of foundational recommendations for TSD and interpreted findings through the Rogerian core conditions of congruence, unconditional positive regard, and accurate empathy.

**Results:**

Findings indicated strong contemporary support for several of recommendations, particularly prioritizing client benefit, maintaining professional boundaries, exercising clinical intentionality, and returning focus to the client. However, ambiguity remained regarding appropriate disclosure content, levels of intimacy, and reasons for disclosure. This ambiguity was most effectively resolved when disclosure decisions were evaluated through relational attunement, client context, and therapeutic intent. TSD also emerged as an important component of culturally responsive practice, particularly when used to facilitate cultural broaching, relational safety, and engagement with issues of power and identity.

**Discussion:**

The findings suggest that the Rogerian core conditions provide a coherent theoretical framework for guiding TSD decision-making while requiring explicit attention to cultural sensitivity and power dynamics. Overall, effective and ethical TSD appears to depend less on specific disclosure characteristics and more on the therapist’s attunement to client needs, therapeutic intent, and relational context. This review contributes a brief person-centered framework to support therapists’ in-the-moment reflective decision-making regarding the ethical, effective, and culturally responsive use of TSD in clinical practice and training.

## Introduction

Therapist self-disclosure (TSD) is a complex and frequently debated intervention within psychotherapy. Defined as the therapist’s intentional sharing of personal information, experiences, or reactions with a client (Hill and Knox, 2002), TSD has been associated with both meaningful therapeutic benefit and potential harm. When used effectively, TSD can enhance therapeutic alliance, foster client engagement, and facilitate corrective emotional experiences (Henretty et al., 2014; Knox and Hill, 2003; Ladany and Lehrman-Waterman, 1999). Conversely, when used inappropriately, TSD carries risks including boundary violations, client discomfort, role reversal, and shifts in focus away from the client’s experience (Zur et al., 2009; Audet, 2011).

Despite decades of research, there remains a lack of consensus regarding when, how, and for what purpose TSD should be used. Existing literature offers broad recommendations—such as prioritizing client benefit, maintaining professional boundaries, and ensuring clinical intentionality (see Table 1; Knox and Hill, 2003)—yet these guidelines often lack the specificity required for real-time clinical decision-making. As a result, therapists are frequently left navigating ambiguity, particularly in emotionally complex or culturally nuanced therapeutic contexts.

**Table 1:** Knox and Hill’s (2003) Suggestions for therapist self-disclosure.

Suggestions for Therapist Self-Disclosure
1. Use therapist self-disclosure because it is a helpful intervention, but use it infrequently and judiciously
2. Use appropriate content in therapist self-disclosure
3. Use appropriate levels of intimacy in therapist self-disclosures
4. Fit the disclosure to the particular client’s needs and preferences
5. Have appropriate reasons for self-disclosing
6. Return the focus to the client after therapist self-disclosure
7. Consider using disclosures of immediacy
8. Consider using disclosures to facilitate termination
9. Ask clients about their responses to therapist self-disclosure
10. Self-disclose about issues that you have mostly resolved rather than those with which you continue to struggle

This ambiguity reflects a deeper ethical tension within clinical practice. On one hand, TSD has demonstrated meaningful therapeutic benefit, supporting the ethical principle of beneficence. On the other, inappropriate disclosure carries the risk of harm, engaging the principle of nonmaleficence. However, emerging literature complicates this dichotomy further, suggesting that non-disclosure—particularly in cross-cultural contexts—may also contribute to harm, including client mistrust, relational disconnection, and the reinforcement of systemic inequities (Lee et al., 2022). Thus, therapists are not choosing between disclosure and neutrality, but rather navigating a complex relational process in which both disclosure and non-disclosure carry potential risks and benefits.

This tension is particularly pronounced among trainees and early-career therapists, who report significant uncertainty regarding the use of TSD. Research has identified concerns related to boundary maintenance, professional identity, and fear of causing harm, alongside a lack of structured training on how to integrate disclosure into practice (Bottrill et al., 2010). Without clear guidance, therapists may either avoid disclosure altogether or engage in it in ways that are misattuned to client needs, increasing the risk of ineffective or harmful use.

The complexity of TSD becomes further amplified within multicultural and socially situated clinical contexts. A central conduit of multicultural competency involves cultural broaching, which is the therapist’s “deliberate and intentional efforts to discuss issues across racial, ethnic and cultural (REC) domains that may impact the client’s present concerns” (Day-Vines et al., 2020, p.107). Emerging literature highlights the role of TSD as a mechanism for cultural broaching, through which therapists explicitly engage with differences in identity, power, and lived experience (Day-Vines et al., 2007; Knox et al., 2019). In these contexts, disclosure may serve to reduce perceived power imbalances, foster relational safety, and promote more authentic therapeutic engagement. At the same time, the absence of such disclosures—particularly when therapists avoid engaging cultural differences—has been identified as contributing to epistemic injustice and the reinforcement of systemic oppression within therapeutic relationships (Lee et al., 2022).

Taken together, these findings highlight a critical gap between empirical knowledge and clinical application. While research has advanced understanding of the risks and benefits of TSD, therapists remain without a coherent, theoretically grounded framework to guide in-the-moment decision-making. Existing recommendations provide valuable direction but do not sufficiently account for the relational, contextual, and cultural complexity inherent in clinical practice. Experts recommend that therapists ground their reasons to disclose, in theory, to safeguard their use of the intervention (Danzer, 2019).

The Rogerian core conditions—congruence, unconditional positive regard, and accurate empathy (Rogers, 1957)—offer a potential resolution to this gap. As foundational principles of person-centered therapy, the core conditions provide a relational framework through which therapists can orient their use of self in the therapeutic process. Rather than prescribing specific behaviors, these conditions emphasize therapist presence, attunement, and responsiveness to the client’s experience. As such, they may offer a coherent and flexible foundation for guiding TSD decision-making, particularly in contexts characterized by uncertainty and complexity.

The purpose of this study is to systematically review contemporary empirical literature on therapist self-disclosure, with a focus on its effectiveness, risks, benefits, and cultural considerations. In addition to synthesizing current findings, this study examines the extent to which existing empirical recommendations align with the Rogerian core conditions as a guiding theoretical framework.

Building on this synthesis, this study contributes a brief person-centered framework designed to support therapists’ in-the-moment decision-making regarding TSD. This framework is intended to bridge the gap between research and practice by offering a clinically applicable approach grounded in relational principles. In doing so, the study also considers implications for therapist training and supervision, with particular attention to the development of practitioner self-awareness and the cultivation of an internalized framework for ethical and effective use of self in therapy.

This study extends existing literature by moving beyond content-based recommendations toward a relational, process-oriented framework for therapist self-disclosure, grounded in the Rogerian core conditions. In doing so, it offers both a theoretical resolution to longstanding ambiguity and a clinically applicable model for in-the-moment decision-making.

## Methods

### Design

This study employed a systematic review methodology to synthesize contemporary empirical literature on therapist self-disclosure (TSD). The review followed Preferred Reporting Items for Systematic Reviews and Meta-Analyses (PRISMA) guidelines to ensure methodological transparency and rigor (Page et al., 2021).

A pragmatic inquiry approach guided the overall design of the study. Pragmatism, as a research paradigm, prioritizes the application of knowledge to real-world contexts, emphasizing what is most useful, workable, and meaningful in practice (Kaushik and Walsh, 2019). This orientation is particularly relevant when examining complex clinical interventions, where variability in context, implementation, and outcomes must be considered (Petticrew et al., 2013). In alignment with this perspective, the present study sought to generate clinically meaningful insights that can support therapist decision-making, rather than to establish prescriptive rules for practice.

### Search strategy

The search of the literature was conducted using multiple databases to ensure a comprehensive synthesis and to reduce bias. The databases accessed included Sage Journals, and the EBSCO research platform, including Academic Search Complete, APA PsycExtra, APA PsycArticles, Psychology and Behavioral Sciences Collection, and APA PsycTherapy. The search terms included *therapist self-disclosure* OR *immediacy* AND *efficacy* OR *effectiveness* OR *impact* OR *benefits* OR *outcomes* OR *risks* OR *adverse effects* OR *benefits* OR *cultural competence* OR *cultural awareness* OR *cultural competency* OR *cultural sensitivity*. These search terms were chosen to include both identified operationalizations of TSD: therapist self-disclosure and immediacy. The search terms were also chosen to capture the range of descriptors in the research on the effectiveness, risks and benefits, and cultural considerations for TSD. The search was limited to peer-reviewed articles published in English within the last 10 years. A 10-year timeframe (2014–2024) was selected to capture contemporary conceptualizations of therapist self-disclosure, including increasing attention to cultural broaching, power dynamics, and culturally responsive practice. The initial search yielded a total of 1,134 articles across the databases.

### Inclusion and exclusion criteria

Studies were included if they:

- Examined therapist self-disclosure within a psychotherapy or counselling context
- Reported empirical findings related to the effectiveness, risks, benefits, or client perceptions of TSD
- Included adolescent or adult client populations
- Were published in peer-reviewed journals

Studies were excluded if they:

- Focused exclusively on client self-disclosure
- Were theoretical papers without empirical data
- Did not directly address TSD within a therapeutic context
- Were not available in English

### Study selection

**Figure 1:**
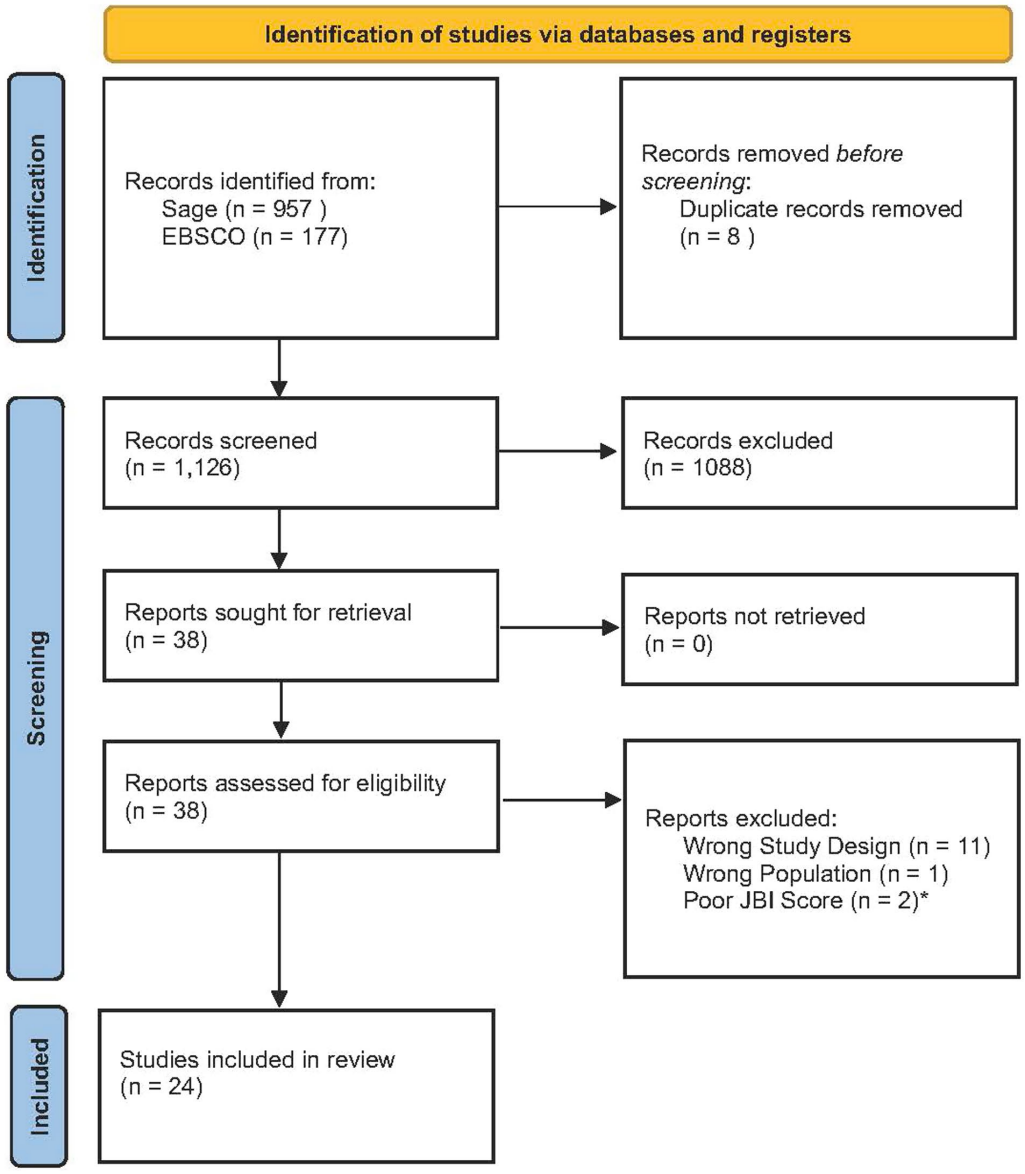
PRISMA flowchart. *Two studies were deemed to have poor methodological integrity with a JBI score of 33.3% and removed from the analysis.

The search results were uploaded to the Rayyan software (Rayyan, 2022), which was used to screen the articles. All identified records were screened in accordance with PRISMA guidelines. Titles and abstracts were initially reviewed for relevance, followed by full-text screening to determine eligibility. The study selection process is illustrated in Figure 1 (PRISMA flow diagram). Following the exclusion of duplicates and an initial screening of the titles and abstracts for applicability (the content was related to TSD), 1,096 articles were excluded. The 38 articles remaining were retrieved and assessed for eligibility. Applying the inclusion and exclusion criteria to the 38 articles, 11 were excluded for an inappropriate study design (conceptual, literature review, etc.), one study was excluded for including an inapplicable population (peer support vs. psychotherapists) and two studies were excluded for poor methodological integrity (JBI score of 33.3%). The final number of articles reviewed under this selection process was 24 (15 qualitative, 7 quantitative, 2 mixed methods), representing a total of 1,785 participant individuals (see Figure 1). For a description of each of the studies reviewed, (see Table 2).

**Table 2:** Description of studies review.

Authors and year of publication	Country	Data Collection	Data Analysis	Participants
Bitar et al. (2014)	USA	In-depth interviews	Phenomenological analysis	10 Mexican-American court-mandated clients
Brownell et al. (2015)	UK	Semi-structured interviews and focus groups	Thematic analysis	37 patients with psychosis receiving positive psychology therapy
Cook et al. (2015)	UK	Semi-structured interviews and focus groups	Constructivist grounded theory	10 participants (patients, victims, Restorative Justice facilitators)
Dunlop et al. (2024)	UK	Q-methodology	Factor Analysis	28 adolescent patients with eating disorders
Elvish et al. (2014)	UK	Semi-structured interviews	Thematic narrative analysis	6 carers of people with dementia
Fuertes et al. (2019)	USA	Actor-partner interdependence modeling	Dyadic ratings	53 therapeutic dyads
Jeffery and Tweed (2015)	UK	Semi-structured interviews	Interpretative phenomenological analysis	8 sexual minority therapists
Kline et al. (2019)	USA	Semi-structured interviews	Consensual qualitative research	21 therapist trainees
Knott et al. (2015)	USA	Quantitative self-report surveys	Functional analytic perspective	80 therapists
Levitt et al. (2016)	USA	Session questionnaires and audio recordings	MCOVA & ANCOVA*	52 therapeutic dyads
Magaldi and Trub (2018)	USA	Qualitative interviews	Grounded theory analysis	21 psychotherapists with varied religious or non-religious backgrounds
Nakamura and Iwakabe (2018)	Japan	Session video recordings with transcripts	Task Analysis	6 Japanese clients
Okun et al. (2017)	USA	Video recordings	Thematic analysis	22 therapeutic dyads (15 interracial, 7 intraracial)
Olofsson et al. (2022)	Norway	Interpersonal process recall	Interpretative phenomenological analysis	6 female inpatients with eating disorders and trauma, 5 therapists
Oyer et al. (2016)	USA	Interviews	Qualitative phenomenological analysis	8 clients with anorexia, 7 therapists
Phiri et al. (2019)	UK	In-depth interviews and focus groups	Thematic analysis	114 participants (15 patients with a psychotic related disorder, 52 laypeople, 22 CBT therapists, 25 mental health practitioners)
Pinto-Coelho et al. (2016)	USA	Session recordings and client ratings	Consensual qualitative research	46 participants (9 therapists, 16 clients, 21 judges)
Pinto-Coelho et al. (2018)	USA	Interviews	Consensual qualitative research	24 participants (13 experienced psychotherapists, 4 interviewers, 7 judges)
Simonds and Spokes (2017)	UK	Surveys	Cross-sectional analysis, serial mediation modeling	120 participants with eating problems
Simonsen and Cooper (2015)	UK	Semi-structured interviews	Interpretative phenomenological analysis	7 former clients of bereavement counselling
Solomonov and Barber (2018)	USA	Online survey	Quantitative analysis	604 clients
Solomonov and Barber (2019)	USA	Online survey	Quantitative analysis	268 therapists
Sunderani and Moodley (2020)	Canada	Semi-structured interviews	Thematic analysis	9 therapists exploring cross-cultural therapist self-disclosure
Ziv-Beiman et al. (2017)	Israel	Randomized Controlled Trial	Regression analysis	86 participants of brief integrative psychotherapy

*Multivariate analysis of covariance, analysis of covariance.

### Data extraction and synthesis

A certain degree of complexity exists in systematically reviewing the topic of TSD. Petticrew et al. (2013) describe that complex interventions can pose challenges for systematic reviews as the characteristics of the intervention may involve multiple components (e.g., disclosures, immediacy), and contextual factors (e.g., client’s and therapist’s intersectionalities, stage of therapist development, ethics) that may impact the intervention, and multiple outcomes (e.g., enhanced rapport, improved outcome, boundary crossings, role reversals, structural oppression of marginalized identities). This complexity is an important consideration guiding the methodological approach and requires the research question be explicitly detailed with a clear research goal. Pragmatism is a recommended research paradigm to address such complex problems, by identifying the continuum-based nature of epistemology and using abductive reasoning that intermixes both deduction and induction (Kaushik and Walsh, 2019). The purpose of pragmatic inquiry is to solve practical problems, recommend action, and provide coherent order to our decision-making processes (Kaushik and Walsh, 2019).

The pragmatic approach to inquiry is deemed applicable to the current research, as it aims to provide actionable direction for therapists’ decision-making process on TSD. Furthermore, pragmatist philosophy is rooted in “a deep sense of justice” and focuses on creating “practical knowledge that has utility for action for making purposeful difference in practice” (Kaushik and Walsh, 2019, p.11). A central aim of the research was to integrate literature on diverse populations’ experience of TSD, equipping therapists with knowledge on the effective use of TSD with marginalized clients.

An inductive and deductive thematic analysis was conducted to synthesize findings. The analysis was informed by Braun and Clarke’s (2006) reflexive thematic analysis approach. Codes were generated through iterative engagement with the data and subsequently organized into themes, which were reviewed and refined through ongoing comparison across studies and consideration of the research objectives. Inductive coding allowed themes to emerge from the data, while deductive analysis examined alignment with the Rogerian core conditions of congruence, unconditional positive regard, and accurate empathy. This dual analytic approach supported both descriptive synthesis and theoretical integration.

**Figure 2:**
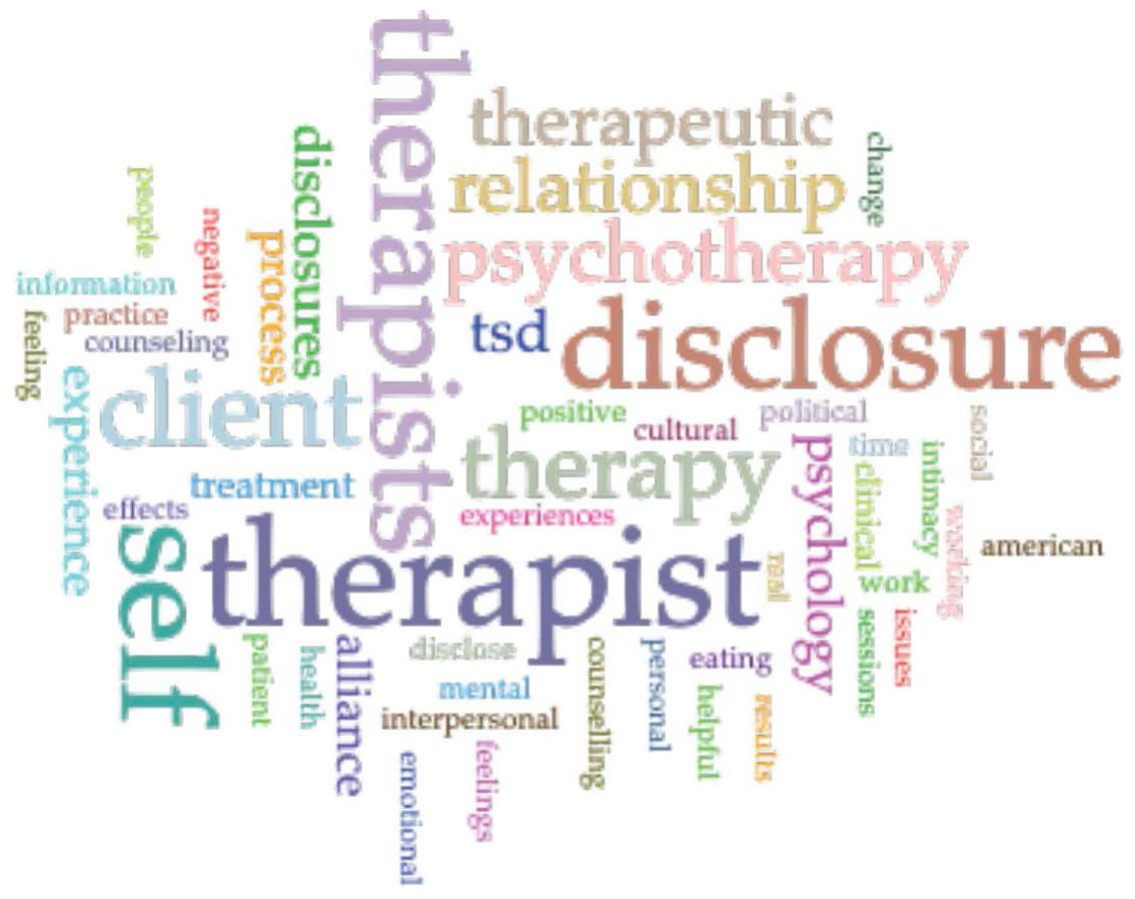
Word cloud generated with Voyant Tools (Sinclair and Rockwell, 2023).

A word frequency visualization (Sinclair and Rockwell, 2023; see Figure 2) was generated to provide a descriptive overview of commonly occurring language within the dataset. This visualization functioned as a supplementary tool to support thematic interpretation, rather than as a primary analytic method.

### Quality appraisal

The methodological quality of included studies was assessed using the Joanna Briggs Institute (JBI) critical appraisal tools (Aromataris and Munn, 2024). Each study was evaluated according to criteria appropriate to its design, including clarity of methodology, rigor of analysis, and validity of conclusions (see Table 3). Findings from the appraisal informed interpretation of the results and supported the inclusion of methodologically sound evidence in the synthesis.

**Table 3:** Methodological integrity analysis table.

Authors and year	Purpose	Authority/credibility	Risk of biases	Measures for reliability	JBI SCORE (%)
Bitar et al. (2014)	“To explore the impact of TSD on the therapeutic alliance within a cross-cultural, mandated treatment context” (p.417)	Marriage and Family Therapists and Faculty of Pfeiffer University, Texas Tech University, and University of Georgia	Sampling, social desirability	Second interviews for clarification, expert peer debriefing	90
Brownell et al. (2015)	To gather feedback from group participants of the positive psychology intervention for psychosis (WELLFOCUS PPT)	Faculty of King’s College London, Institute of Psychiatry, support from the National Institute for Health and Research	Researcher, social desirability	Demonstrated reflexivity (2 coders, team discussions to address discrepancies)	80
Cook et al. (2015)	To explore the psychological processes involved in Restorative Justice (RJ) interventions to generate a theoretical model for RJ in forensic mental health service	Faculty of King’s College London, Institute of Psychiatry, support from the National Institute for Health and Research	Sampling, response	Demonstrated reflexivity (independent coders, team discussion and supervision)	80
Dunlop et al. (2024)	To explore adolescents perceptions on what influences their decision to disclosure in treatment for eating disorders (ED)	Faculty of Department of Psychological Interventions, School of Psychology, University of Surrey	Researcher	Q-sorts were research derived	80
Elvish et al. (2014)	To explore the meaning of and process of change in psychotherapy from the perspective of carers of people with dementia	Faculty of University of Manchest, School of Nursing, Midwifery and Social Work	Sampling	Multiple coders and several readings	80
Fuertes et al. (2019)	To explore the relationship between therapists’ and clients’ attachment style and the real relationship and treatment progress, as well as the influence of TSD on attachment style and the real relationship and the attachment style	Gordon F Derner School of Psychology, Adelphi University	Social desirability	Standardized measures with high internal consistency and test–retest reliability, exploratory factor and principal components analysis	80
Jeffery and Tweed (2015)	To explore the experiences of LGB mental health practitioners’ experiences of disclosing their sexual orientation to clients	Trainee clinical psychologist, and clinical psychologist and faculty at the Universities of Staffordshire and Keele	Sampling, researcher	Reported and demonstrated use of reflexivity	100
Kline et al. (2019)	To examine psychodynamic-interpersonal therapist trainee’s experiences of alliance ruptures	Faculty of Department of Counseling, Higher Education, and Special education, University of Maryland, Department of Psychology, University of Maryland	Social desirability	Demonstrated reflexivity, expert review of interview protocol, use of external auditor, trained coding team	100
Knott et al. (2015)	To examine intimacy and self-disclosure in therapists’ personal and therapeutic contexts	Faculty of University of Houston and Rogers Memorial Hospital	Self-report, social desirability, sampling	Standardized measures with adequate to excellent internal consistency	75
Levitt et al. (2016)	To explore the frequency and types of TSDs as related to outcome and alliance	Faculty of Department of Psychology University of Massachusetts, Northwestern University, The Center for Discovery, Mental Illness Research, Education, and Clinical Center, Department of Veterans Affairs Capitol Health Care Network, University of Memphis	Researcher, confirmation	Standardized measures, trained coders, consensus in categorization	100
Magaldi and Trub (2018)	To understand how therapists are disclosing about spirituality and religion with clients, to determine the efficacy of therapy from largely non-religious therapists treating religious/spiritual clients	Faculty of Department of Counseling at Lehman College, City University	Sampling, researcher	Reported and demonstrated use of reflexivity throughout (bracketing conversations, thick descriptions of biases)	100
Nakamura and Iwakabe (2018)	To delineate essential components of corrective emotional experiences (CEE) through study of client and therapists behaviors and interactions in session	Faculty of the Human Science Division of Ochanomizu University	Researcher, sampling	Demonstrated reflexivity (research derived and clearly described task model, two experimenters, reported grounding inferences)	100
Okun et al. (2017)	To examine processes of engagement in first sessions of therapy with therapists of color and non-Latino White patients	Faculty of Department of Psychology, The New School for Social Research	Researcher, sampling	Efforts to ensure homogeneity of between groups of dyads, demonstrated reflexivity (trained and diverse coders, structured discussions of biases)	100
Olofsson et al. (2022)	To uncover how Eating Disorder (ED) non-responders with childhood trauma (CT) experience the working alliance during in-session processes	Faculty of the Department of Psychology, University of Oslo, Forskningsveien	Researcher, sampling, self-report	Use of standardized measures, demonstrated reflexivity (bracketing and reflective discussions)	100
Oyer et al. (2016)	To examine therapist factors to aid the working alliance in working with clients with Anorexia Nervosa	Faculty of the Department of Counseling Psychology University of Northern Colorado	Sampling, recall, social desirability	Two independent researchers analyzed transcriptions	80
Phiri et al. (2019)	To identify how client-initiated self-disclosure may influence the therapeutic alliance with Black and minority ethnic populations with schizophrenia	Faculty of University of Southampton, and Southern Health NHS Foundation Trust	Researcher	Reflexivity, cross-checking, triangulation of participant responses, independent review and expert consultation	100
Pinto-Coelho et al. (2016)	To investigate TSD in open-ended psychodynamic psychotherapy	Faculty of the Department of Psychology, College of Behavioral and Social Sciences, University of Maryland	Sampling, researcher	21 independent judges, naturalistic setting, bracketing methods, standardized measures	100
Pinto-Coelho et al. (2018)	To learn experienced therapists views on the helpfulness and use of successful TSDs, unsuccessful TSDs, and how to manage the urge to disclose, and advice for trainees	Faculty of the Department of Psychology, College of Behavioral and Social Sciences, University of Maryland	Sampling, self-report	Empirically based interview questions, piloted, trained judges, use of external auditors, bracketing	100
Simonds and Spokes (2017)	To model relationships between types of TSD, therapeutic alliance, patient self-disclosure, shame, and severity of eating problems	School of Psychology, University of Surrey, Guildford, UK	Sampling	Some standardized measures, some empirically based non-standardized measures	75
Simonsen and Cooper (2015)	To deepen understanding of client perceived helpful practices for bereavement	Maggie’s Centre, Aberdeen, UK & Counselling Unit, University of Strathclyde, Glasgow, UK	Sampling, self-report	Reflexivity, clearly documented methodology, cross checking, used direct quotes	100
Solomonov and Barber (2018)	To understand how political polarization effects patients experiences in therapy	Faculty of The Derner School of Psychology, Adelphi University	Self-report, recall, sampling	Standardized measure with large and somewhat diverse sample	75
Solomonov and Barber (2019)	To explore therapists’ perspectives on political TSD, perceived shared values with clients, and the therapeutic alliance	Faculty of The Derner School of Psychology, Adelphi University	Self-report, recall, sampling	Standardized measure with large and somewhat diverse sample	75
Sunderani and Moodley (2020)	To examine therapists’ perceptions of TSD during cross-cultural therapy, to examine how to adapt therapy skills for minority populations	Faculty of Department of Psychology at Univeristy of Guelph-Humber, and Department of Applied Psychology & Human Development at OISE, University of Toronto	Response	Verified speech-to-text transcriptions, participant review of transcripts, demonstrated reflexivity (reflective commentary and debriefing sessions)	100
Ziv-Beiman et al. (2017)	To examine the effect of immediate and non-immediate TSD in brief integrative psychotherapy for mild to moderate distress	Faculty of Tel Aviv-Yaffo Academic College, Tel Aviv University	Researcher, self-report	Ecologically valid, controlled manipulation of TSD, homogeneity across experiment groups, standardized measure with satisfactory internal consistency	91.67

The JBI score is based on the JBI manual for evidence synthesis (Aromataris and Munn, 2024).

### Reflexivity and researcher positioning

Given the use of a pragmatic inquiry approach, the analytic process was shaped by the researcher’s perspectives, values, and clinical orientation (Braun and Clarke, 2006; Kaushik and Walsh, 2019). To support methodological rigor, several strategies were implemented to mitigate potential bias. Clear inclusion and exclusion criteria were established to reduce selection bias, and a reflexive journaling process was used throughout data collection and analysis to support bracketing of assumptions and reactions. This process enabled ongoing awareness of how personal perspectives may shape interpretation of the data.

The researcher approaches knowledge from a humanistic and person-centered perspective, grounded in principles of dignity, autonomy, and self-actualization (Gandhi and Mukherji, 2023). Consistent with person-centered theory, the researcher was trained to incorporate an awareness of herself in the therapeutic experience and to communicate that experience to the client if appropriate (Rogers, 1961). The researcher has noted the profound relational deepening in therapeutic relationships through disclosing with clients and perceives such transparency as an integral component for establishing trust and safety in the clinical context. While the researcher holds the intervention of therapist self-disclosure in high regard, she has also experienced uncertainty with some personal disclosures and observed moments of disconnection with clients following a therapist disclosure. To address potential theoretical and clinical bias, thematic analysis was used to examine whether the Rogerian core conditions emerged inductively within the literature, rather than being imposed *a priori*. Findings were subsequently evaluated to determine the extent to which the Rogerian conditions meaningfully captured the implications of the studies reviewed.

As a sole investigator, the study is further shaped by the researcher’s social location and interpretive lens. The researcher occupies both privileged and marginalized social positions, which informed a reflexive stance toward issues of culture, identity, and power within the literature. Reflexive practices were used to support critical engagement with these dynamics throughout the analytic process.

### Data availability statement

The datasets generated and analyzed during the current study, including the PRISMA flow diagram and detailed tables of included studies are provided as supplementary material.

## Results

The Voyant Tools software (Sinclair and Rockwell, 2023) was used to a create a word cloud, depicting the most common words of the 24 articles included in the systematic review (see Figure 2). Notably, the words *relationship, interpersonal, personal, intimacy,* and *alliance* were all identified as some of the most common used. The frequency of these words suggests a common theme amongst the literature, primarily that the use of TSD relates to the therapeutic relationship. Additional themes identified in the word cloud include *cultural, political, positive, negative, helpful, change, real, feelings, emotional,* and *eating*. The relevance of these words is further explored through the interpretative thematic analysis described below.

The data of the studies were extracted and synthesized utilizing the MAXQDA24 software (VERBI Software, 2024) to support thematic organization and analysis. Thematic analysis revealed four primary themes: (1) TSD as a mechanism for relational attunement, (2) the tension between therapeutic benefit and potential harm, (3) TSD as a tool for cultural broaching and navigating power dynamics, and (4) the limits of content-based guidance.

### TSD as a mechanism for relational attunement

Across studies, TSD was consistently associated with enhancing relational depth when used with intentionality and attunement to client needs. Findings indicated that disclosure can foster trust, deepen connection, support client engagement, humanize the therapist, and reduce shame or isolation when aligned with the client’s emotional state and therapeutic goals (Dunlop et al., 2024; Henretty et al., 2014; Knox and Hill, 2003; Ladany and Lehrman-Waterman, 1999; Levitt et al., 2016; Ziv-Beiman et al., 2017). This relational function of TSD was further reflected in qualitative findings, where therapists described disclosure as a means of “demonstrating compassionate care” and fostering connection through shared humanity (Berg et al., 2017).

These findings are consistent with foundational recommendations outlined by Knox and Hill (2003), which emphasize intentionality and client benefit in guiding therapist self-disclosure. Rather than functioning as a discrete intervention, TSD appeared to operate relationally, contributing to therapist authenticity and presence within the therapeutic alliance.

For example, disclosures motivated by compassionate care and normalization have been identified as central reasons therapists use TSD, particularly when disclosure helps clients feel less alone or less ashamed in their experience (Berg et al., 2017; Pinto-Coelho et al., 2018). Similarly, disclosures of immediacy, in which therapists name their present experience within the therapeutic relationship, have been associated with relational deepening, client openness, and enhanced engagement (Hill et al., 2018; Ziv-Beiman et al., 2017).

The TSD also appeared to support relational attunement by humanizing and deformalizing the therapist. In several studies, disclosures that allowed clients to experience the therapist as a real person were associated with greater trust, perceived genuineness, and a more collaborative therapeutic relationship (Dunlop et al., 2024; Audet, 2011; Fuertes et al., 2019; Levitt et al., 2016). In some clinical contexts, therapist self-disclosure was described as “almost obligatory” for maintaining the therapeutic alliance. For example, when working with clients experiencing delusions and paranoia, disclosure was identified as “almost the only action able to preserve the alliance” (Phiri et al., 2019, p. 14), highlighting the role of disclosure in sustaining relational safety under conditions of mistrust. These findings suggest that the effectiveness of TSD is less dependent on the specific content disclosed and more closely related to the therapist’s capacity for attunement, timing, and responsiveness within the relational context.

### The tension between therapeutic benefit and potential harm

Despite its relational benefits, TSD was also associated with potential risks when used without sufficient clinical judgment. Studies identified concerns including boundary crossings, role reversals, client discomfort, progressive over-disclosure, and shifts in focus away from the client (Audet, 2011; Jowers et al., 2019; Peterson, 2002; Zur et al., 2009).

These concerns align with Knox and Hill’s (2003) recommendation to use “appropriate content” and avoid disclosures that may be clinically disruptive. However, the current literature reveals a more nuanced and, at times, contradictory picture of what constitutes “appropriate” disclosure.

For example, disclosures of therapist anxiety have been identified as potentially problematic when they arise from the therapist’s own discomfort and fail to return focus to the client (Jowers et al., 2019). Similarly, disclosures of current or recent personal challenges have been rated less favorably than disclosures of distant or resolved experiences, supporting caution around unresolved material (Moody et al., 2021; Pinto-Coelho et al., 2018). Disclosures that communicate difference between therapist and client may also create distance when the disclosure highlights dissimilarity in ways that leave the client feeling misunderstood or less connected (Levitt et al., 2016).

At the same time, findings suggest that the impact of such disclosures is highly context-dependent. Some disclosures traditionally approached with caution may contribute to increased client trust, perceived authenticity, and therapeutic engagement when they are brief, purposeful, and clearly connected to the client’s process. Thus, rather than supporting a fixed distinction between appropriate and inappropriate content, the literature suggests that the clinical impact of TSD is shaped by timing, intent, relational context, and therapist attunement.

These findings extend Knox and Hill’s (2003) framework by demonstrating that while general guidelines regarding appropriateness are useful, they do not fully capture the contextual and relational complexity inherent in clinical decision-making.

### TSD as a tool for cultural broaching and navigating power dynamics

A prominent theme within the literature was the role of TSD in culturally responsive practice. In particular, TSD was identified as a mechanism for cultural broaching, enabling therapists to acknowledge and engage differences and similarities in identity, power, and lived experience (Bitar et al., 2014; Day-Vines et al., 2007; Jeffery and Tweed, 2015; Knox et al., 2019; Lee et al., 2022).

In these contexts, disclosure functioned to reduce perceived power imbalances, foster relational safety, communicate openness to discussing identity, and support more authentic therapeutic engagement. Multicultural TSD was associated with many of the same therapeutic functions as general TSD, including fostering the therapeutic relationship, increasing trust, normalizing client experience, modelling disclosure, and humanizing the therapist (Bitar et al., 2014; Jeffery and Tweed, 2015; Okun et al., 2017; Phiri et al., 2019).

The reviewed literature also highlighted the significance of shared identity and lived experience. Therapists reported greater comfort using TSD with clients who shared similar cultural backgrounds, and clients sometimes selected therapists based on salient shared identities, such as religion, gender, or cultural background (Magaldi and Trub, 2018; Sunderani and Moodley, 2020). Disclosures of shared identity were associated with increased trust, safety, acceptance, and depth of therapeutic work, though authors also cautioned that shared-identity disclosures may risk minimizing the client’s unique experience or creating unrealistic expectations of understanding (Baumann et al., 2020; Bennett and Clark, 2021; Magaldi and Trub, 2018; Phiri et al., 2019).

Findings also revealed important tensions in cross-cultural contexts. Therapists were more inclined to disclose cultural similarities than differences, despite evidence suggesting that avoiding difference may reinforce systemic power imbalances within the therapeutic relationship (Magaldi and Trub, 2018; Sunderani and Moodley, 2020). In contrast, disclosures that respectfully acknowledged difference or positionality were identified as potentially important for establishing credibility, trust, and a more egalitarian alliance, particularly where power differences were accentuated (Bitar et al., 2014; Jeffery and Tweed, 2015; Lee et al., 2022).

The literature also suggests that client-initiated questions may function as tests of therapist trustworthiness, especially among clients from marginalized communities (Phiri et al., 2019). In such moments, the therapist’s comfort, openness, and responsiveness may be more clinically meaningful than the specific content disclosed (Phiri et al., 2019). These findings suggest that TSD may function not only as a relational tool, but also as a mechanism for promoting cultural safety and addressing epistemic inequities, while requiring careful contextual sensitivity.

### The limits of content-based guidance

Despite broad agreement regarding the goals of TSD, the literature revealed a persistent limitation of content-based recommendations. Studies varied considerably in how they defined appropriate disclosure, and findings repeatedly demonstrated that the same disclosure could be experienced as either beneficial or harmful depending on context, timing, relational factors, and therapist intent. This suggests that ambiguity may not stem from insufficient guidelines, but from the inherently contextual nature of TSD itself.

This ambiguity was reflected in variability across studies in how TSD was defined, conceptualized, and evaluated. For example, recommendations regarding moderate levels of intimacy, minimal detail, or disclosure of resolved rather than current concerns have received some support, yet remain insufficiently explored empirically across naturalistic clinical contexts (McCormick et al., 2019; Moody et al., 2021; Pinto-Coelho et al., 2018; Ziv-Beiman, 2013).

The limits of content-based guidance were especially evident in relation to “appropriate” content. The literature cautions against disclosures that may shift focus to the therapist, create role reversal, or highlight dissimilarity in ways that weaken connection. Yet the same categories of disclosure—personal experience, current reactions, identity, values, or difference—may also strengthen trust, credibility, and therapeutic alliance when used with attunement and clear clinical purpose.

The results further suggest that therapists must reflect not only on their reasons for disclosing, but also on their reasons for not disclosing. In cultural contexts, avoiding disclosure may be clinically prudent in some cases, particularly where disclosure risks imposing therapist beliefs or shifting focus away from the client. However, avoidance may also reflect therapist discomfort and may leave important aspects of identity, power, or difference unaddressed (Magaldi and Trub, 2018; Jeffery and Tweed, 2015).

The persistence of this ambiguity suggests that while Knox and Hill (2003) provide an important foundational framework, their recommendations do not fully resolve the complexity therapists encounter in moment-to-moment practice. This gap between empirical knowledge and clinical application highlights the need for a structured yet flexible framework to support therapists in the use of self within the therapeutic relationship.

## Discussion

This study sought to synthesize contemporary empirical literature on therapist self-disclosure (TSD) and to examine the extent to which existing recommendations provide meaningful guidance for clinical practice. Consistent with prior research, findings indicate that TSD can function as a powerful and effective therapeutic intervention when used with intentionality and attunement. At the same time, the results highlight a persistent and clinically significant gap: therapists are left without clear, operationalized guidance for navigating TSD in real-time practice.

Interpretation of therapist self-disclosure varies considerably across theoretical orientations. Historically, psychodynamic approaches have often emphasized therapist restraint and neutrality, whereas humanistic and experiential traditions have viewed therapist transparency and use of self as central therapeutic processes. More contemporary relational, multicultural, and integrative approaches tend to emphasize contextual responsiveness rather than fixed rules regarding disclosure. While these differing perspectives may contribute to ongoing debate regarding TSD, the findings of this review suggest that relational attunement, client benefit, and therapeutic intentionality emerge as common considerations across modalities.

Across the literature, TSD emerges as a mechanism not only for therapeutic connection, but for navigating power, identity, and lived experience within the therapeutic relationship. In multicultural contexts, disclosure may function as a form of cultural broaching, supporting relational safety and reducing perceived power imbalances. Conversely, the absence of disclosure—particularly around identity or positionality—may contribute to mistrust, relational disconnection, and epistemic injustice, in which clients’ experiences are inadvertently minimized or misunderstood.

Across themes, TSD was consistently found to function as a process of relational responsiveness rather than a discrete technique. Its impact appears to be shaped less by the content of the disclosure itself and more by the interaction of therapist intention, timing, relational context, and responsiveness to the client. This challenges the prevailing emphasis within existing guidelines on categorizing disclosures as “appropriate” or “inappropriate.” While such distinctions offer a degree of ethical scaffolding, they do not sufficiently capture the complexity of clinical reality.

Notably, the literature demonstrates that disclosures traditionally regarded as inappropriate—such as sharing current emotional experiences, acknowledging value differences, or revealing aspects of the therapist’s ongoing life—may, in certain contexts, contribute meaningfully to therapeutic engagement. This is particularly evident in culturally responsive and relationally complex work, where such disclosures may foster authenticity, support cultural safety, and strengthen the therapeutic alliance. These findings suggest that rigid adherence to content-based rules may obscure the more critical determinants of therapeutic impact, namely relational attunement, client experience, and contextual sensitivity.

At the same time, the risks associated with TSD remain substantial. Boundary crossings, role reversals, and progressive over-disclosure underscore the ethical weight of this intervention. Importantly, the literature also highlights the often-overlooked risks of non-disclosure (Lee et al., 2022; Magaldi and Trub, 2018; Jeffery and Tweed, 2015). Taken together, these findings position TSD within a broader ethical and relational tension in psychotherapy, in which both disclosure and non-disclosure carry the potential for benefit and harm.

### The Rogerian core conditions as a guide to clinical decision making

The findings of this study suggest that the Rogerian core conditions—congruence, unconditional positive regard, and accurate empathy (Rogers, 1957)—offer a coherent theoretical framework for understanding and navigating the ambiguity inherent in therapist self-disclosure. Rather than prescribing specific behaviors, the core conditions provide a relational orientation that aligns closely with the factors identified in the literature as central to effective TSD.

Congruence reflects the therapist’s capacity to engage authentically, allowing disclosure to emerge from a genuine and integrated internal experience. Congruence, in this context, is experienced by the client not simply as therapist honesty, but as relational transparency that enhances trust and reduces perceived distance. However, the literature demonstrates that authenticity alone is insufficient. Disclosures that are congruent but not attuned to the client’s needs—such as those driven by therapist anxiety—may contribute to confusion or emotional burden rather than therapeutic benefit.

Unconditional positive regard ensures that therapist self-disclosure is experienced by the client as fundamentally non-judgmental, affirming, and grounded in acceptance. The literature consistently highlights that the therapeutic value of disclosure is closely tied to whether the client experiences the therapist as *prizing* them—communicating care, respect, and an absence of evaluation (Rogers, 1957, 1995). Several findings suggest that TSD may function as a mechanism for reducing client shame. Disclosures grounded in compassionate intent and normalization have been shown to help clients feel less alone, less defective, and more deeply understood in their experience (Berg et al., 2017; Pinto-Coelho et al., 2018). In this way, TSD does not merely communicate information, but relationally conveys acceptance of the client’s humanity.

From a person-centered perspective, this reflects a temporary “removal of the clinical mask,” in which the therapist’s disclosure communicates not comparison or evaluation, but shared humanity. When unconditional positive regard is present, disclosure is experienced as connecting rather than distancing—deepening the client’s sense of being known without fear of judgment. Conversely, when this condition is absent, even well-intentioned disclosures may be experienced as subtly evaluative, misattuned, or distancing, particularly in clients who are sensitive to criticism, marginalization, or relational rupture.

Accurate empathy supports the therapist’s ability to anticipate and respond to the client’s experience of disclosure. The literature underscores that the same disclosure may be experienced as validating by one client and misattuned by another, depending on relational, developmental, and cultural factors. As such, empathy is essential in ensuring that disclosure enhances, rather than disrupts, the client’s sense of being understood.

Taken together, these conditions provide theoretical navigation to the ambiguity identified in the literature. Rather than attempting to define “appropriate” disclosure content, the core conditions shift the focus toward the relational experience created by the disclosure—specifically, whether it fosters a sense of safety, understanding, and non-judgment for the client.

### A person-centered framework for in-the-moment clinical decision-making

Building on this theoretical foundation, this study contributes a brief person-centered framework designed to support therapists in the in-the-moment use of TSD. While the preceding section explains why the Rogerian conditions theoretically resolve ambiguity, the following framework operationalizes these principles into practical reflective questions. The framework invites therapists to engage in three interrelated evaluative questions, grounded in the core conditions:

(1) Congruence: Where is this disclosure coming from?

Therapists are encouraged to reflect on the origin of the impulse to disclose. For example, the literature highlights instances in which therapists disclose personal experiences in response to their own anxiety or discomfort. While such disclosures may be authentic, they may not serve the client’s process. This step supports therapists in differentiating between disclosures that arise from relational presence and those that are driven by internal pressure or need.

(2) Unconditional Positive Regard: How might this disclosure be experienced?

This domain emphasizes whether the disclosure communicates acceptance, care, and non-judgment. Therapists are invited to consider whether the disclosure will help the client feel valued and understood, or whether it may introduce subtle evaluation, comparison, or expectation. This is particularly important in contexts where clients may already feel vulnerable to judgment or marginalization.

(3) Accurate Empathy: Is this responsive to this client, in this moment?

Therapists are encouraged to consider the client’s emotional state, relational history, and cultural context when determining whether to disclose. For example, disclosures of personal struggle may be experienced as deeply validating by some clients, while overwhelming or distracting for others. This step reinforces the need for moment-to-moment attunement.

Together, this framework provides a structured yet flexible process that reflects both the empirical findings of this review and the relational principles of person-centered theory. It supports therapists in engaging in a continuous process of reflection and attunement, allowing for responsive and ethically grounded use of self-disclosure in practice.

### Implications for clinical practice and training

A central implication of this study is that competence in the use of TSD cannot be reduced to knowledge of guidelines alone. Rather, it requires the development of an internalized capacity for self-reflection, attunement, and ethical discernment—which can be understood as an internalized, moment-to-moment reflective process—what may be thought of as an “internal supervisor.” This internal evaluation process has been identified in person-centered therapists’ reported experiences of using TSD, to help guide whether to disclose as well as determine their intentions for disclosing (Jolley, 2019). This internal supervisory process enables therapists to evaluate their use of self in real time, particularly in moments of uncertainty or emotional activation. Training programs may benefit from shifting away from rule-based instruction toward approaches that cultivate this capacity, including the development of self-awareness, emotional regulation, and relational sensitivity.

Supervision also plays a critical role in this process. Providing space for therapists to reflect on their experiences of disclosure—including moments of uncertainty, misattunement, or perceived rupture—supports the development of clinical judgment and ethical responsiveness.

Importantly, training must also address the cultural and epistemic dimensions of TSD. As the findings of this study suggest, disclosure may function as a necessary component of culturally responsive practice. Developing competence in TSD therefore requires not only relational skill, but also the ability to engage with power, identity, and difference in ways that promote cultural safety and relational equity. In practice, this shifts the clinician’s task from deciding what to disclose to discerning how disclosure will be experienced within the therapeutic relationship.

A notable and clinically significant gap identified in this review is the limited empirical attention given to the process of returning focus to the client following therapist self-disclosure. While Knox and Hill (2003) explicitly recommend that therapists redirect the conversation back to the client, the present synthesis found minimal direct investigation into how this process occurs in practice or how it influences therapeutic outcomes. However, indirect evidence within the reviewed literature suggests that failure to return focus may contribute to role reversal, client burden, and therapeutic drift. For example, trainee disclosures driven by therapist anxiety have been shown to maintain focus on the therapist’s internal experience rather than the client’s process (Jowers et al., 2019), highlighting how easily the relational center of therapy can shift.

In contrast, clinical examples within the literature demonstrate that effective TSD is often characterized by brevity and immediate reintegration of the client’s experience. For instance, disclosures used to normalize client experience are most therapeutically effective when followed by an invitation for the client to elaborate on their own meaning-making (Patmore, 2020).

These findings suggest that the “return of focus” may represent a critical yet underdeveloped component of safe and effective TSD. As such, training programs may benefit from shifting emphasis away from solely *what* to disclose toward *how to transition out of disclosure*, supporting therapists in maintaining the primacy of the client’s experience.

### Limitations and future directions

Several limitations should be considered when interpreting the findings of this study. As a systematic review guided by a pragmatic inquiry approach, the analysis was shaped by the researcher’s interpretive lens and prioritization of clinically relevant themes. While reflexive strategies were employed to support rigor, the findings remain influenced by the researcher’s perspectives and theoretical orientation.

In addition, variability in how TSD was defined and measured across studies limited the ability to draw definitive conclusions regarding specific practices. The reliance on predominantly Western-based research may also constrain the generalizability of findings across diverse cultural contexts.

Future research would benefit from more targeted empirical investigation into the conditions under which specific types of disclosure are experienced as helpful or harmful. Further exploration of TSD within diverse cultural and clinical populations is also warranted, particularly in relation to power, identity, and relational dynamics. Future research may explore the role of demographic congruence, including age and gender similarities between therapists and clients, in shaping disclosure processes and outcomes. Future research may also explore the applicability of self-disclosure frameworks among other healthcare professions, including physicians, nurses, and rehabilitation professionals, where relational dynamics similarly influence care.

Future quantitative syntheses may be possible as the literature continues to mature and more consistent definitions and measures of therapist self-disclosure emerge. At present, considerable variability exists in how self-disclosure is conceptualized, operationalized, and measured across studies, limiting opportunities for broad meta-analytic investigation. More narrowly focused reviews examining specific forms of disclosure, client perceptions, or therapeutic alliance outcomes may provide promising avenues for future quantitative analysis.

Continued development and evaluation of this person-centered guide for therapist self-disclosure is necessary to assess the utility and effectiveness of the framework in maximizing the therapeutic benefits and mitigating the risks of this important intervention. Lastly, while the present study reviewed the literature through a person-centered framework, there may be another theoretical modality that more accurately and effectively encompasses the empirical literature on therapist self-disclosure. Additional studies are needed to explore guidelines for the intervention through alternative theoretical groundings.

## Conclusion

Therapist self-disclosure remains one of the most debated interventions in psychotherapy, yet the findings of this systematic review suggest that efforts to identify universally appropriate or inappropriate disclosures may be inherently limited. Across studies, the impact of disclosure was shaped less by its content than by the relational context in which it occurred, the therapist’s intention, and the client’s experience of the intervention.

Rather than resolving the complexities of therapist self-disclosure through increasingly specific rules, this review proposes a shift toward relationally grounded clinical judgment. Drawing on Rogers’ core conditions of congruence, unconditional positive regard, and empathy, the proposed framework offers clinicians a way of approaching disclosure decisions that is responsive to context while remaining anchored in enduring therapeutic principles.

Ultimately, competent use of therapist self-disclosure may depend not on what therapists choose to reveal, but on their capacity for self-awareness, relational attunement, and ethical discernment. Supporting the development of these capacities through training, supervision, and reflective practice represents an important direction for both clinical practice and future research.

## Data Availability

The original contributions presented in the study are included in the article/supplementary material, further inquiries can be directed to the corresponding author.
